# Exploring urine:serum fractional excretion ratios as potential biomarkers for lupus nephritis

**DOI:** 10.3389/fimmu.2022.910993

**Published:** 2022-08-24

**Authors:** Samar A. Soliman, Samantha Stanley, Kamala Vanarsa, Faten Ismail, Chi Chiu Mok, Chandra Mohan

**Affiliations:** ^1^ Department of Rheumatology & Rehabilitation, Faculty of Medicine, Minia University, Minia, Egypt; ^2^ Department of Biomedical Engineering, University of Houston, Houston, TX, United States; ^3^ Department of Medicine, Tuen Mun Hospital, Hong Kong, Hong Kong SAR, China

**Keywords:** biomarker, lupus nephritis, fractional excretion, ALCAM, platelet factor-4, properdin

## Abstract

**Objectives:**

The goal of this exploratory study is to determine if urine:serum fractional excretion ratios can outperform the corresponding urinary biomarker proteins in identifying active renal disease in systemic lupus erythematosus (SLE).

**Methods:**

Thirty-six adult SLE patients and twelve healthy controls were examined for serum and urine levels of 8 protein markers, namely ALCAM, calpastatin, hemopexin, peroxiredoxin 6 (PRDX6), platelet factor 4 (PF4), properdin, TFPI and VCAM-1, by ELISA. Fractional excretion of analyzed biomarkers was calculated after normalizing both the urine and serum biomarker levels against creatinine. A further validation cohort of fifty SLE patients was included to validate the initial findings.

**Results:**

The FE ratios of all 8 proteins interrogated outperformed conventional disease activity markers such as anti-dsDNA, C3 and C4 in identifying renal disease activity. All but VCAM-1^FE^ were superior to the corresponding urine biomarkers levels in differentiating LN activity, exhibiting positive correlation with renal SLEDAI. ALCAM^FE^, PF4^FE^ and properdin^FE^ ratios exhibited the highest accuracy (AUC>0.9) in distinguishing active LN from inactive SLE. Four of the FE ratios exhibited perfect sensitivity (calpastatin, PRDX6, PF4 and properdin), while ALCAM^FE^, PF4^FE^ and properdin^FE^ exhibited the highest specificity values for active LN. In addition, several of these novel biomarkers were associated with higher renal pathology activity indices. In the validation cohort ALCAM^FE^, PF4^FE^ and properdin^FE^ once again exhibited higher accuracy metrics, surpassing corresponding urine and serum biomarkers levels, with ALCAM^FE^ exhibiting 95% accuracy in distinguishing active LN from inactive SLE.

**Conclusions:**

With most of the tested proteins, urine:serum fractional excretion ratios outperformed corresponding urine and serum protein measurements in identifying active renal involvement in SLE. Hence, this novel class of biomarkers in SLE ought to be systemically evaluated in larger independent cohorts for their diagnostic utility in LN assessment.

## Introduction

Systemic lupus erythematosus (SLE) is a complex chronic autoimmune disease distinct from other organ-specific autoimmune disorders in terms of its wide spectrum of presentation and accumulation of manifestations over time, making diagnosis difficult, especially at the early stage. In SLE patients, lupus nephritis (LN) is a prominent cause of morbidity and mortality. LN affects the overall quality of life, beginning with mild adverse effects on daily activities, ability to work and eventually progressing to total impairment as end-stage renal disease (ESRD) sets in (1–3). A real challenge has been the finding of a less invasive substitute to renal biopsy, the gold standard for LN diagnosis and classification. Despite being feasible for routine follow-up, current LN laboratory screenings lack both sensitivity and specificity for detecting active renal lesions in SLE (4–6). Consequently, the pursuit for superior biomarkers that can detect and predict renal disease flares in SLE patients remains pressing.

Recently, focus on urine (u) biomarkers in LN has grown as they appear to be more appealing and practical than serum (s) markers since they originate directly from the source of inflammation. In contrast to conventional biomarker discovery studies, high-throughput proteomics-based approaches such as electrochemiluminescence (ECL) immunoassays, aptamer-based assays, antibody-based microarrays and mass spectrometry fuel the discovery of new biomarker candidates in an unbiased manner *via* comprehensive screening of large numbers of molecules, not only those already known to be involved in pathogenesis of the disease (7, 8).

Stanley et al. (8) investigated more than 1000 distinct proteins in the urine of active LN patients of different ethnicities, using an aptamer-based approach. This comprehensive screening revealed several proteins that were considerably higher in active LN urine compared to inactive SLE urine. Based on Ingenuity Pathway Analysis, the elevated proteins clustered into multiple biological pathways including interleukins, chemokines, TNF/TNF-receptor superfamily members, proteins fundamental for extracellular matrix turnover and/or fibrosis, metalloproteases, and cadherins (8). Among the urinary proteins that performed best in distinguishing and correlating with renal disease activity with high sensitivity and specificity were activated leukocyte cell adhesion molecule (ALCAM), calpastatin, hemopexin, peroxiredoxin-6 (PRX6), platelet factor-4 (PF-4), properdin, tissue factor pathway inhibitor (TFPI), and vascular cell adhesion protein-1 (VCAM-1).

The origin of the elevated urinary proteins in LN is uncertain as they may potentially be serum derived; alternatively, they may relate to increased renal expression and secretion into the urine or result from reduced tubular reabsorption due to cell injury. Here, we assess the diagnostic utility of serum and urine levels of the same biomarker candidates, as well as the relative enrichment of the biomarker protein in the urine compared to the serum, calculated using fractional excretion (FE) rates, as described (9, 10). Specifically, we explore the use of FE rates of selected proteins instead of absolute urinary levels of biomarkers in LN, and whether they exhibit enhanced diagnostic performance compared to creatinine-normalized urine biomarkers using a well-phenotyped SLE cohort with concurrent serum and urine samples.

## Patients and methods

### Patients

Initially, serum and urine samples were obtained from thirty-six consecutive adult patients (≥18 years of age) fulfilling ≥4 of the 1997 American College of Rheumatology (ACR) classification criteria for SLE (11), who attended to the rheumatology outpatient clinics or when hospitalized inpatient at the Tuen Mun Hospital, Hong Kong, China. Biosamples from an additional fifty SLE patients were included as a validation cohort, again drawn from the same clinics. The study was approved by the Ethics Committees of Tuen Mun hospital and the University of Houston. All enrolled patients completed a written informed consent based on good clinical practice and the Declaration of Helsinki. Demographic, clinical data and conventional measures of disease activity were collected prospectively. Serum and urine samples from twelve healthy individuals of comparable sex and age were included as controls.

### Assessment of SLE disease activity and flares

SLE disease activity was assessed using SLEDAI-2K, a validated tool in clinical practice and research (12). Renal activity was assessed using the renal domain scores of SLEDAI (range 0–16; 0 = inactive LN). At enrollment, patients were categorized into 3 groups; active renal SLE (LN, patients with renal SLEDAI score ≥ 4), active non-renal SLE (patients with total clinical SLEDAI ≥1, but renal SLEDAI=0) and inactive SLE (patients with total clinical SLEDAI = 0, asymptomatic with no findings of organ activity, subclinical hypocomplementemia and/or elevated autoantibodies allowed). The physician’s global assessment (PGA) of disease activity of SLE (range 0–3) (13) was performed by the attending physician to score his/her impression of the patient’s disease activity at the time of assessment.

### Serum and urine biomarker assays

Serum and urine samples were prepared, aliquoted and frozen at -80°C within 2 hours of sample collection. Biomarker levels in serum and urine were assayed using human enzyme-linked immunosorbent assay (ELISA) kits. ALCAM (Cat.#:DY656), PF-4 (Cat.#:DY795), TFPI (Cat.#:DY2974), VCAM-1 (Cat. #:DY809) were assayed using ELISA kits from R&D Systems (Minneapolis, Minnesota, USA). Calpastatin (Cat. #:MBS075904) was assayed using an ELISA kit from MyBioSource, Inc. (San Diego, California, USA), whereas Hemopexin (Cat.#:E-80HX), Peroxiredoxin 6 (Cat. #: ab187406) and Properdin (Cat. #:LS-F6408-1) were assayed using an ELISA kit from Immunology Consultants (Portland, OR, USA), Abcam (Waltham, MA, USA), and LifeSpan BioSciences, Inc. (Seattle, WA, USA), respectively, according to the manufacturer’s manual. Optical densities at 450 nm were measured using a microplate reader ELX808 (BioTek Instruments, Winooski, VT) and sample concentrations were calculated using a standard curve. All measurements were assayed in duplicate. Serum samples were diluted 1:2 (Calpastatin, properdin and TFPI), 1:5 (PF-4), 1:10 (VCAM-1), 1:50 (ALCAM and hemopexin), while Peroxiredoxin 6 serum assays was not diluted. Urine samples were diluted 1:50 (both ALCAM and hemopexin), 1:5 (PF-4), 1:2 (both properdin and TFPI), 1:10 (VCAM-1), while for calpastatin and peroxiredoxin 6 assays, the urine was not diluted, as detailed before (8). These dilutions were selected to ensure all biomarker concentrations were within the linear range of the assays. The assay range for the different biomarkers are detailed in **Supplementary Table 2**. The values of urinary protein markers were normalized to urine creatinine. Performers/readers of biomarker assays were blinded to patient groups and clinical information. Fractional excretion (FE) ratios of the protein markers in urine relative to the serum were calculated using the following equation, the same way fractional excretion of sodium is calculated:

Fractional Excretion (FE)=(Urine biomarker/urine Cr) ÷ (Serum biomarker/serum Cr)

### Renal histology

The renal histopathologic features of the active LN patients were analyzed using a kidney biopsy performed by a nephropathologist. LN classification, histologic signs of active inflammation, and features of chronicity or degenerative damage associated with LN were determined using the International Society of Nephrology/Renal Pathology Society (ISN/RPS) criteria (14). In accordance with the National Institute of Health’s LN recommendations, renal pathology activity and chronicity were assessed using biopsy activity and chronicity indices (AI, CI, respectively). Numeric values were assigned to each of the activity and chronicity component variables, which were then summated to calculate the AI score (range 0-24; 0= no LN activity) and CI score (range 0-12; 0= no LN chronicity) (15). The time interval between the renal biopsy and serum/urine procurement was 2-4 weeks.

### Statistical analysis

Continuous variables were expressed as mean ± standard error of the mean (SEM) or as medians with interquartile range (IQR) and range, while percentages are displayed for categorical variables. Comparison of values among different groups was performed using the non-parametric Mann Whitney U-tests. Pearson’s correlation coefficient was used for correlation analysis of continuous and normally distributed data. Otherwise, the nonparametric Spearman’s correlation coefficient was used. Rho values between 0.2–0.4 were considered weak; 0.4-0.6, modest; >0.6 strong. A two-tailed *P* value less than 0.05 was considered statistically significant.

The diagnostic accuracy of fractional excretion of each biomarker as well as conventional markers of SLE were assessed using receiver operating characteristic curve (ROC) analysis, and the corresponding area under the curve (AUC; range 0–1) was calculated. ROC analysis was also used to ascertain the sensitivity, specificity, and optimal cut-off values. The criteria for improved performance by a FE ratio biomarker was increased ROC AUC accuracy value, with statistical significance of equal or higher value, compared to that of the corresponding urine biomarker. All statistical analyses were performed using GraphPad Prism v.6.0 (GraphPad, San Diego, CA, USA).

## Results

### Clinical characteristics of study population

A total of 36 SLE patients (94.4% females) and 12 healthy controls were included in the cross-sectional study, where concurrent serum and urine samples were available from the same patients. Their mean age was 42.5 ± 17.14 years. The mean SLEDAI score of the patients was 11.2 ± 9.0, ranging from 2 to 42. Based on SLEDAI assessment, 12 patients were categorized as active renal SLE (active LN), 12 patients were active non-renal and 12 patients were classified as having clinically inactive SLE. 12 healthy female subjects, age and sex matched (100% females, mean age 44.7± 6.3) served as controls. The validation cohort included 25 active LN patients and 25 inactive SLE patients, drawn from the same outpatient clinics, not overlapping with the subjects used for the initial study.

Clinical characteristics of the studied SLE patients are summarized in **Table 1**. The total SLEDAI and PGA scores were significantly higher in active renal patients compared to those with non-renal and inactive SLE. The SLICC organ damage scores, however, were comparable among the 3 groups of SLE patients. Prednisolone, hydroxychloroquine, and azathioprine were used by patients in the 3 groups, while mycophenolate mofetil (MMF) was significantly more frequently used by patients with active renal SLE.

**Table 1:** Demographic and clinical characteristics of SLE patients.

	Active renal SLE(N=12)	Active non-renal SLE (N=12)	Inactive SLE(N=12)	*P-value
Mean ± SEM; Median (IQR); N (%)				
**Age, years**	41.1 ± 17.8	37.4 ± 16.8	48.9 ± 16.2	0.25
**Females**	12 (100)	11(91.7)	11(91.7)	0.59
**SLE duration, years**	3.8 ± 6.0	3.2 ± 5.0	11.1 ± 5.6	0.003
**Clinical disease activity**				
Neuropsychiatric	3 (25)	1 (8.3)	0 (0)	0.14
Musculoskeletal	6 (50)	7 (58.3)	0 (0)	0.006
Renal	12 (100)	0 (0)	0 (0)	<0.001
Mucocutaneous	2 (16.7)	9 (75)	0 (0)	0.004
Serositis	2 (16.7)	2 (16.7)	0 (0)	0.33
Hematological	3 (25)	10 (83.3)	0 (0)	0.001
**Laboratory assessment**				
Anti-dsDNA titer	208.4 ± 30.02	186.3 ± 33.03	125.8 ± 31.08	0.228
Anti-Sm positive	3 (25)	4 (33.3)	3 (25)	0.934
Anti-Ro positive	3 (25)	6 (50)	2 (16.7)	0.097
Anti-La positive	4 (33.3)	5 (41.7)	5 (41.7)	0.978
Complement C3 (mg/dl)	60.7 ± 4.6	65.4 ± 3.5	98.4 ± 3.8	< 0.001
urine PCR, mg/mg	3.44 ± 0.77	0.12 ± 0.08	0.11 ± 0.09	< 0.001
**SLEDAI Score**	19.1 ± 9.8	11.2 ± 3.9	2.7 ± 1.1	<0.001
- Renal SLEDAI score†	8(4-16)	0	0	<0.001
**PGA score**	2.2 ± 0.2	1.8 ± 0.3	0.3 ± 0.2	<0.001
**SLICC damage score**	1.5 ± 1.4	1.1 ± 1.4	0.7 ± 0.5	0.48
**Medications**				
Prednisolone	10 (83.3)	10 (83.3)	6 (50)	0.25
Prednisolone dose	40 ± 21.9	17.8 ± 7.7	5 ± 2.2	<0.001
Hydroxychloroquine	8 (66.7)	10 (83.3)	7 (58.3)	0.54
Azathioprine	6 (50)	8 (66.7)	5 (41.7)	0.62
Cyclophosphamide	2 (16.7)	1 (8.3)	0 (0)	0.39
Mycophenolate mofetil	7 (58.3)	2 (16.7)	1 (8.3)	0.02
Cyclosporin A	1 (8.3)	2 (16.7)	0 (0)	0.39
Tacrolimus	4 (33.3)	1 (8.3)	1 (8.3)	0.21

IQR, Interquartile range; PCR, Protein: Creatinine ratio; PGA, physicians’ global assessment; SLE, systemic lupus erythematosus; SEM, standard error of mean; SLEDAI, SLE disease activity index; SLICC, SLE international collaborative clinic. †: Range 0-16; 0 = inactive LN.

*P: comparison among the three groups.

Among the active renal SLE group, median renal SLEDAI score was 8 (range 4-16). The uPCR concentrations ranged from 0.3 to 10.5 mg/mg, with significant increase in active renal patients compared to active non-renal and inactive SLE (P<0.001). In 12 (100%), 10 (83.3%), and 3 (25%) patients, respectively, pyuria, hematuria, and active urinary casts were present. A kidney biopsy was conducted in all 12 active LN patients. **Table 2** illustrates the histopathologic features in this group. ISN/RPS LN classes III and VI were identified in 4 (33.3%) patients each, ISN/RPS classes II and V in 1 (8.3%) patient each, and mixed class LN (III+V or IV+V) in 2 (16.6%) patients. Renal pathology activity and chronicity were also assessed in these biopsies (**Table 2**), with a median biopsy activity index of 7 (range 2-11) and chronicity index of 3 (range 0-6).

**Table 2:** Histopathologic features of the active lupus nephritis patients.

ISN/RPS classification (n=12)	
- Class II, N (%)	1 (8.33)
- Class III, N (%)	4 (33.33)
- Class IV, N (%)	4 (33.33)
- Class V (pure), N (%)	1 (8.33)
- Mixed class III/IV+V, N (%)	2 (16.66)
**Histopathologic features**	
***Activity Index, median (IQR)§***	*7 (2-11)*
- Endocapillary proliferation score >0, N (%)	7 (58.33)
- Glomerular WBC infiltration score >0, N (%)	5 (41.66)
- Hyaline deposits score >0, N (%)	4 (33.33)
- Karyorrhexis score >0, N (%)	3 (25)
- Cellular crescents score >0, N (%)	3 (25)
- Interstitial inflammation score >0, N (%)	4 (33.33)
***Chronicity Index, median (IQR)*** *¶*	*3 (0-6)*
- Glomerulosclerosis score >0, N (%)	3 (25)
- Fibrous crescents score >0, N (%)	1 (8.33)
- Tubular atrophy and interstitial fibrosis scores >0, N (%)	1 (8.33)

ISN/RPS: International Society of Nephrology/Renal Pathology Society

§: Range 0-24; 0 = no LN activity features, ¶: Range 0-12; 0 = no LN chronic changes.

### Serum and urine levels of assayed biomarkers

**Figure 1:**
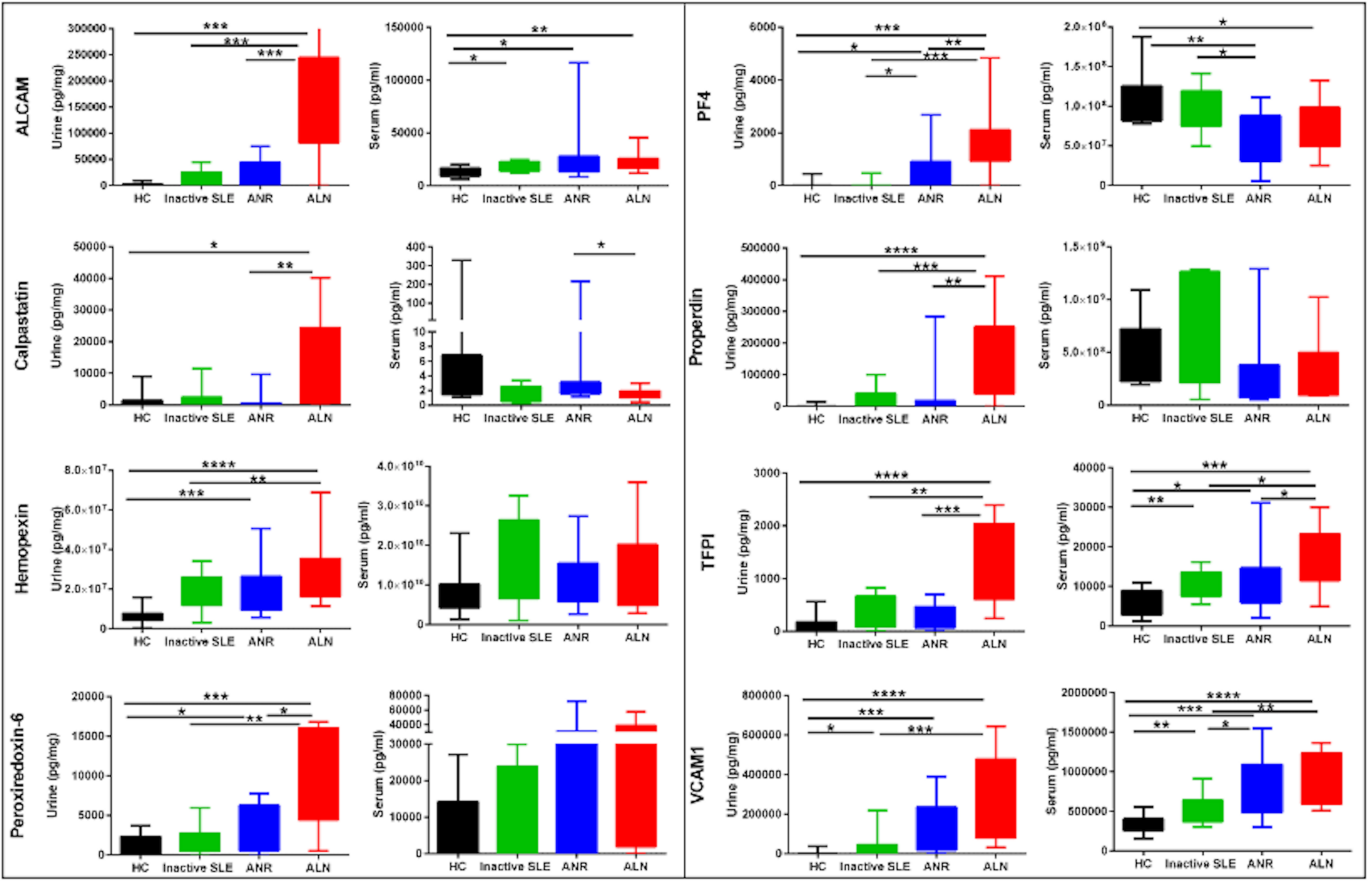
Serum and urine levels of 8 protein biomarkers in SLE patients and controls. The Y axes show the values of the 8 studied biomarkers (inter-quartile and range), while the X axes display the 4 groups interrogated (12 healthy controls; 12 inactive SLE; 12 active non-renal SLE; 12 active renal disease(LN)). Urinary biomarker levels were normalized to urinary creatinine. Within each set of plots, the urine profiles are plotted to the left (Cr normalized), while the serum profiles are presented to the right. The urine biomarker data has been reported previously (8). * = P<0.05, ** = P<0.01, *** = P<0.001, **** = P<0.0001.

**Figure 2:**
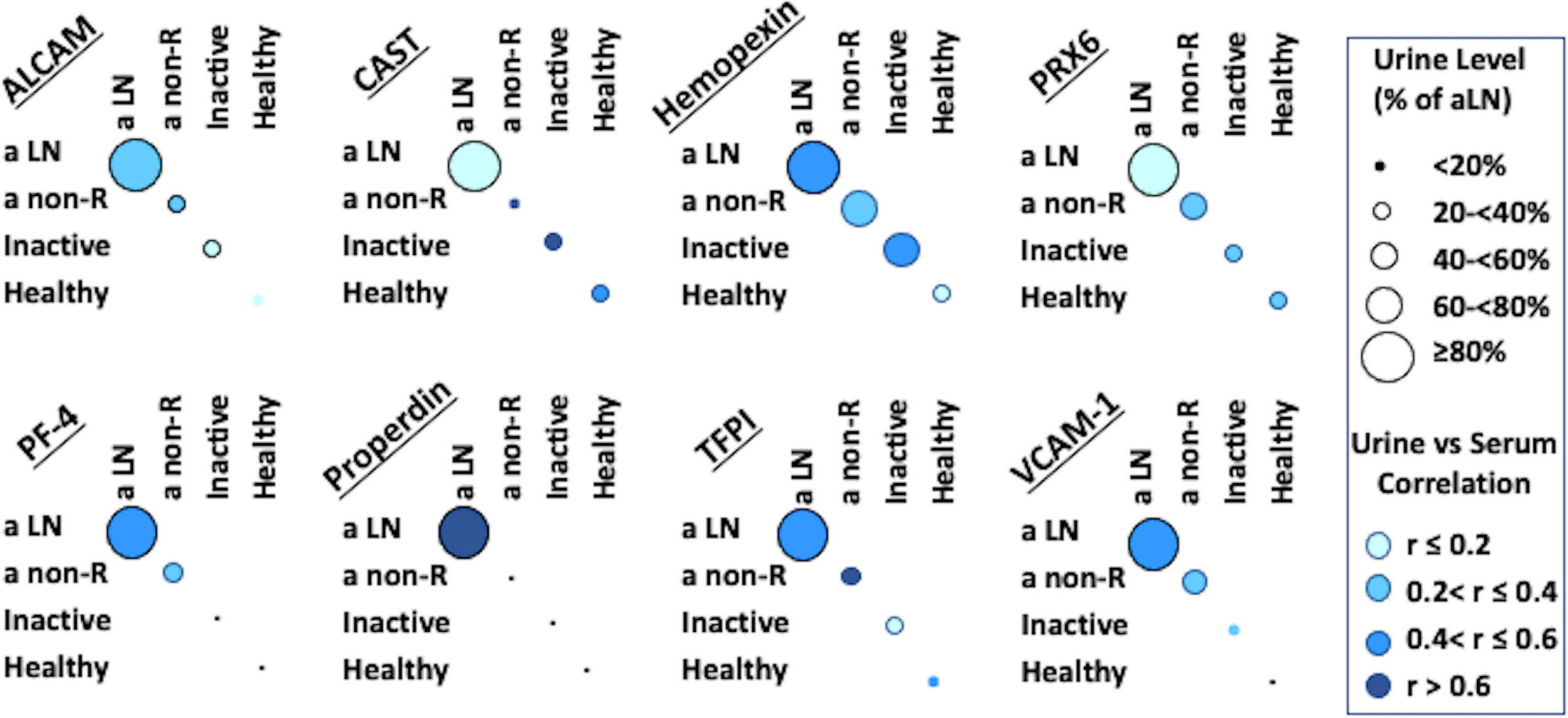
Correlation of Serum and Urine protein biomarkers in SLE patients and controls. For each subject, the corresponding values of the protein in urine and serum were considered, in order to ascertain any correlation between these 2 measures. Intensity of blue color represents the strength of serum/urine correlations in each of the 4 subject groups examined, while the size of the circles represents urine biomarker level in each group compared to the level in the active LN group; in all cases the biomarker level in active LN was set to be 100%, corresponding to the maximal circle size.

Serum and urine levels of the eight tested biomarker proteins among the three groups of SLE patients as well as healthy controls, as assayed by ELISA, are illustrated in **Figure 1**. Urine levels of all biomarker proteins assayed were highest among active LN patients, as previously reported (8). Levels of uALCAM, u PRX6, uPF4, uProperdin and uTFPI protein markers were significantly higher in patients with active renal disease than active non-renal disease, inactive SLE or healthy controls. uCalpastatin levels showed significant difference between active renal and active non-renal disease subjects, as well as controls. Both uHPX and uVCAM-1 exhibited significantly higher levels in active renal compared to inactive SLE and controls, as reported (8). However, the serum levels of the assayed proteins from the same patients were not significantly different between active renal SLE patients and inactive SLE controls, with the exception of VCAM-1 (**Figure 1**). Interestingly, the urine levels of the biomarker proteins correlated with the serum levels of the same proteins, particularly in active LN patients, weakly (ALCAM), modestly (Hemopexin, PF-4, TFPI, VCAM-1) or strongly (properdin), as plotted in **Figure 2**, even though the serum levels could not distinguish active LN from other SLE patients, with most of the biomarkers.

### Fractional excretion ratios of assayed biomarkers

**Figure 3:**
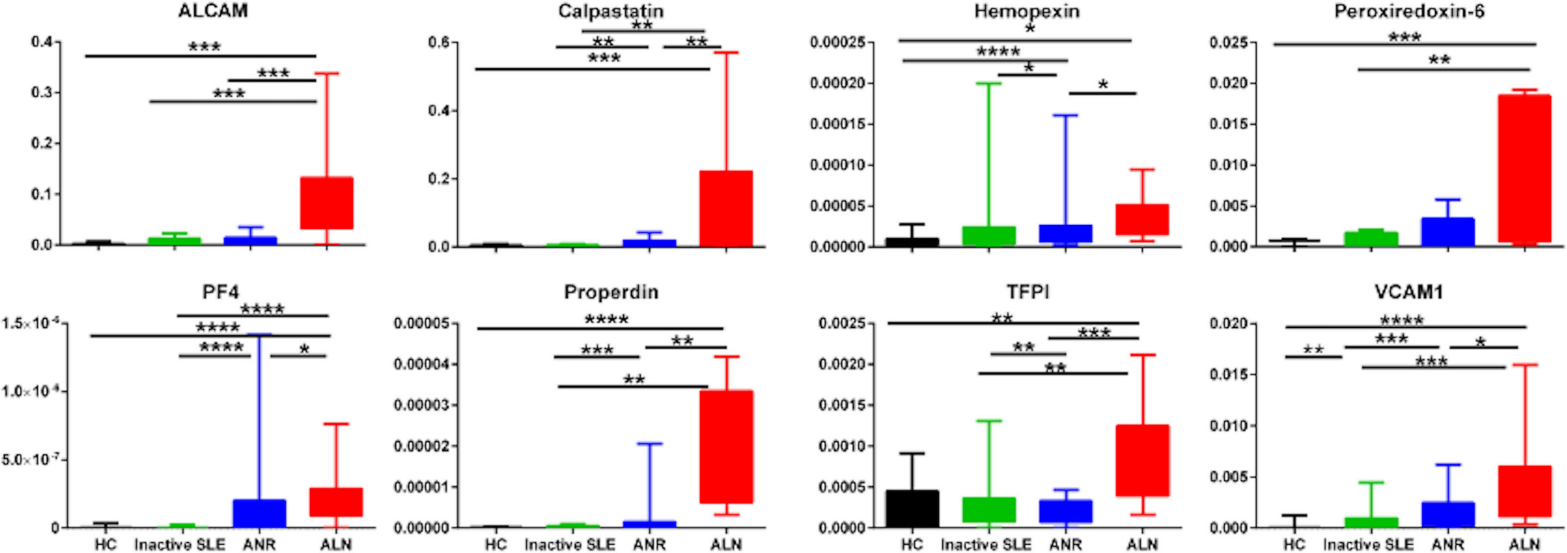
Fractional Excretion ratios of eight protein biomarkers in SLE patients and controls. Fractional excretion (FE) ratios of all 8 biomarker proteins were calculated by dividing the urine biomarker levels by the corresponding serum biomarker levels, after normalizing both against urine or serum creatinine, respectively. The Y axes show the values of the fractional excretion ratios of studied biomarkers (inter-quartile and range), while the X axes display the 4 groups interrogated (12 healthy controls; 12 inactive SLE; 12 active non-renal disease; 12 active renal disease). * = P<0.05, ** = P<0.01, *** = P<0.001, **** = P<0.0001.

To assess if the biomarker proteins were enriched in the urine relative to the serum, we next calculated the fractional excretion ratios of these biomarkers, by dividing the urine biomarker levels by the corresponding serum biomarker levels, after normalizing both against urine or serum creatinine, respectively. **Figure 3** shows the fractional excretion ratios of the eight biomarker proteins measured in the four groups of subjects studied. Fractional excretion ratios of all biomarkers were highest in active LN patients. ALCAM^FE^, calpastatin^FE^, PF4^FE^, properdin^FE^, TFPI^FE^ and VCAM-1^FE^ markers were significantly increased in active LN patients than those with active non-renal disease, inactive SLE or healthy controls. Hemopexin^FE^ was significantly higher in active LN compared to active non-renal as well as controls, PRX6^FE^ while showed significant difference between active renal, inactive SLE and controls.

### Correlation of biomarker fractional excretion ratios with SLE conventional disease activity parameters

**Figure 4:**
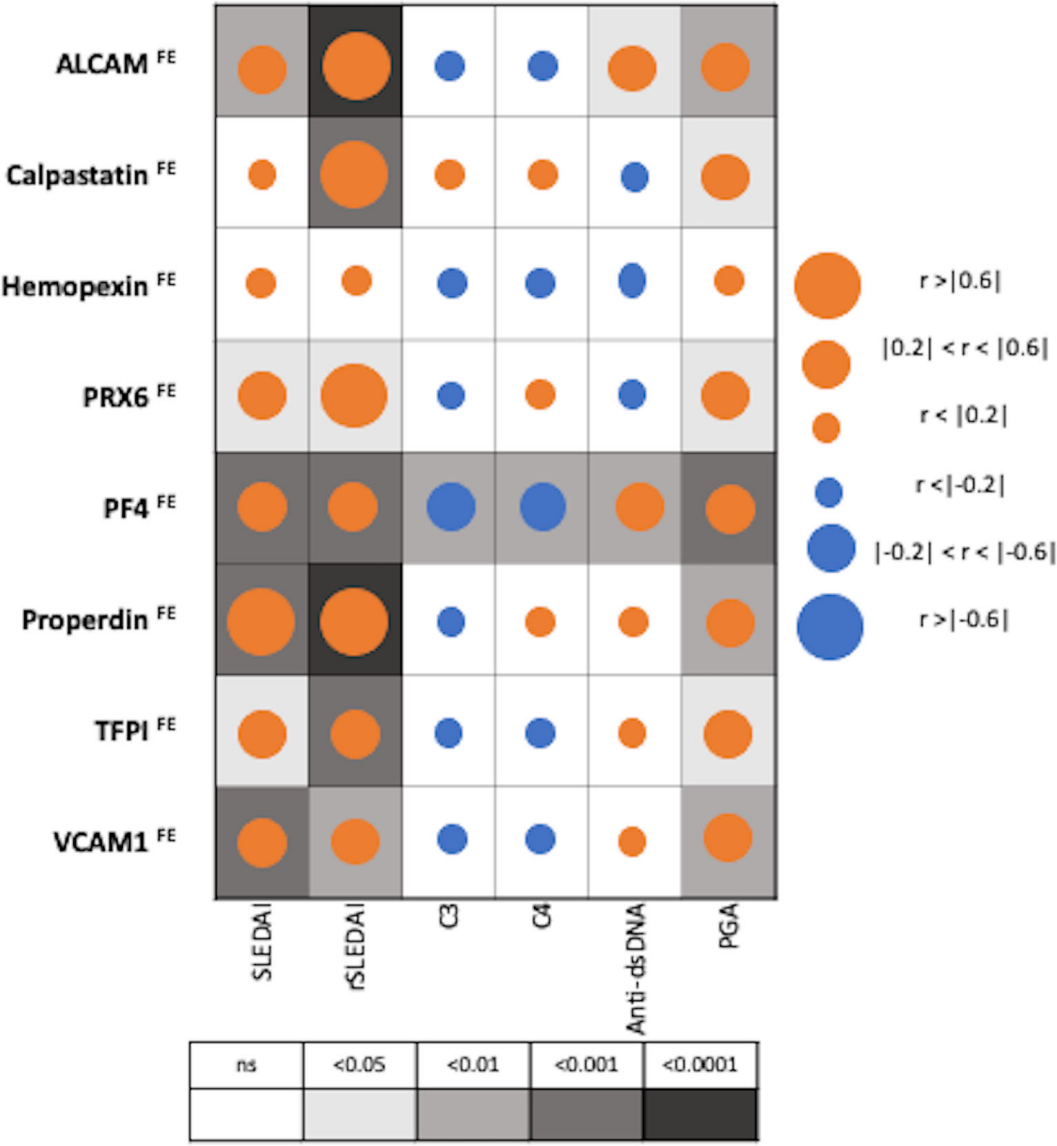
Correlation of Fractional Excretion (FE) ratios of eight protein biomarkers with conventional disease activity measures in SLE. The derived FE ratios of the eight interrogated biomarkers were examined for their correlation with SLEDAI, renal-SLEDAI, C3, C4, anti-dsDNA and Physician Global Assessment (PGA) values. Positive and negative correlations are denoted by orange and blue circles respectively, while statistical significance is denoted using grey-scale boxes. Overall, the urine biomarker fractional excretion ratios correlate best with renal-SLEDAI in LN.

Among patients with SLE (N=36), PF4^FE^ correlated significantly with all conventional disease activity parameters including SLEDAI, renal SLEDAI, PGA, complement C3, C4 and anti-dsDNA, while hemopexin^FE^ did not correlate with any of disease activity parameters, as shown in **Figure 4**. Renal SLEDAI scores exhibited the best correlation coefficients with FE ratios of the assayed biomarkers, showing strong significant correlation with properdin^FE^ (Rho 0.79, P< 0.0001), followed by ALCAM^FE^ (Rho 0.64, P<0.0001), calpastatin^FE^ (Rho 0.64, P<0.001), and PRX6^FE^ (Rho 0.64, P 0.01). PF4^FE^, TFPI^FE^ and VCAM-1^FE^ values showed “good” significant correlations with renal SLEDAI (Rho 0.58, 0.58, 0.49 respectively). The biomarkers’ FE correlations with SLEDAI scores were significant but weaker compared to those with renal SLEDAI, with only properdin^FE^ displaying a strong significant correlation (Rho 0.65, P<0.001). TFPI^FE^ correlated weakly with SLEDAI (Rho 0.37, P<0.05). Apart from hemopexin^FE^, all other biomarker FE ratios significantly correlated positively with PGA score.

Only PF4^FE^ exhibited significant inverse correlation with complement C3 and C4 values [(Rho -0.43, P<0.01), (Rho -0.44, P<0.01)] respectively, while anti-dsDNA values were significantly correlated with ALCAM^FE^ (Rho 0.35, P<0.05) and PF4^FE^ (Rho 0.44, P<0.01), as shown in **Figure 4**.

In this small cohort, since all but 2 of the subjects had proliferative LN, we were not able to assess if the interrogated biomarkers were predictive of LN class. Importantly, several FE ratios (but not the corresponding urine protein levels) were higher in LN patients with high AI (≥7) compared to patients with lower AI (0–6), including ALCAM^FE^ (p=0.03), properdin^FE^ (p=0.02), PF4^FE^ (p=0.06) and hemopexin^FE^ (p=0.07), with the former two attaining statistical significance and the latter two barely missing significance (using a 2-tailed t-test), despite the relatively small sample size. In contrast, complement C3, C4, anti-dsDNA and uPCR were not significantly different in patients with higher AI or CI. In addition, hemopexin^FE^ correlated strongly with the renal biopsy chronicity index (Pearson correlation r = 0.65; p = 0.023).

### Comparing the diagnostic performance of urine:serum FE ratios in LN against conventional and emerging biomarkers

**Figure 5:**
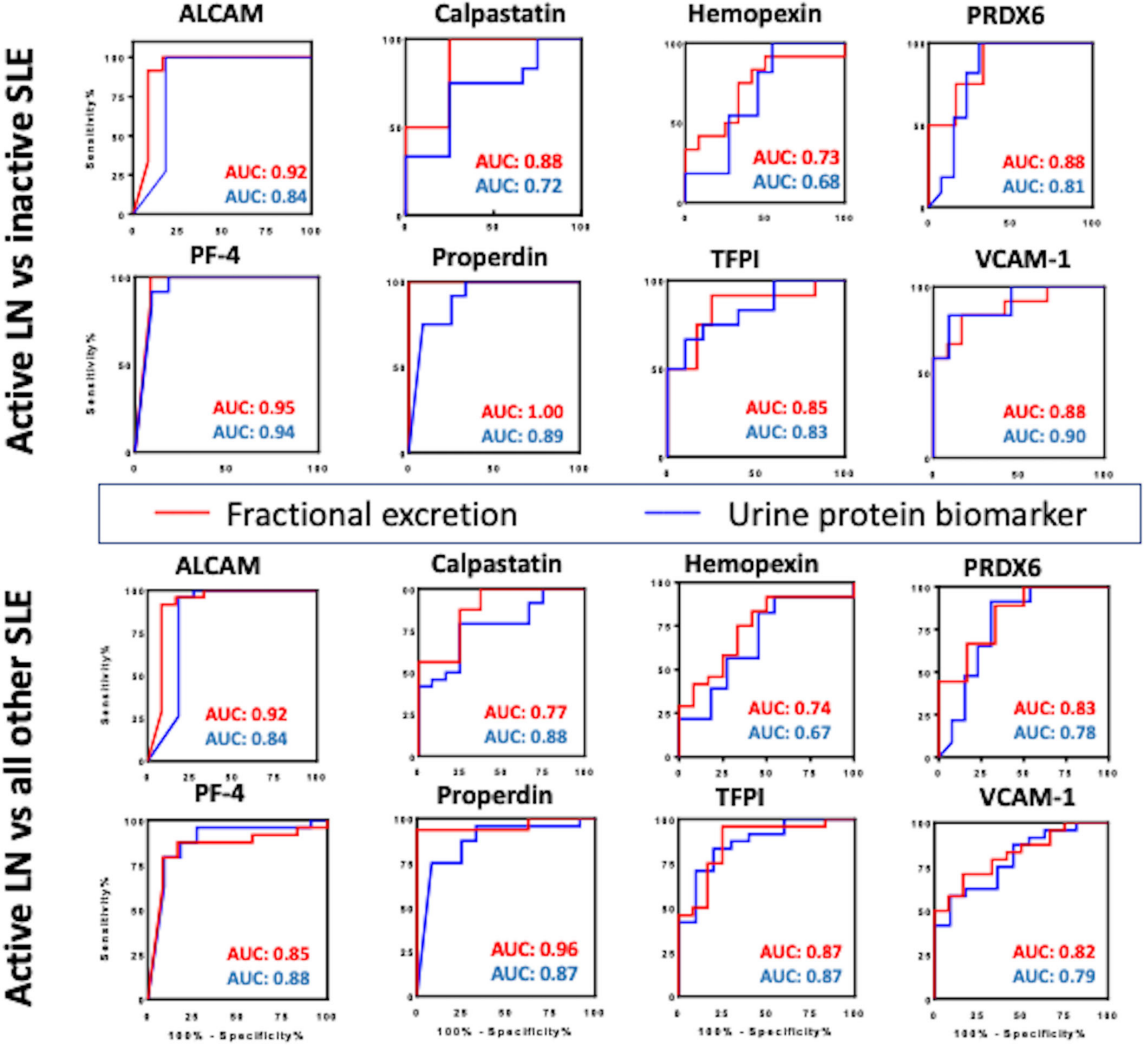
Comparing the diagnostic potential of FE ratios and their corresponding urine biomarkers in identifying active LN. Receiver Operating Characteristic (ROC) curves were generated for the 8 urine protein markers (blue line) and their corresponding FE ratios (red line) in differentiating active lupus nephritis from inactive SLE (top) and active lupus nephritis from all other SLE patients (active non-renal and inactive SLE) (bottom). The 95% confidence intervals, sensitivity and specificity values for these markers are detailed in **Table 2**.

To compare the diagnostic performance of the urine:serum fractional excretion ratios of the eight biomarkers assayed against the performance of the creatinine-normalized urinary biomarkers, ROC analysis was performed. As detailed in **Table 3** and **Figure 5**, three FE biomarkers (ALCAM^FE^, PF4^FE^ and properdin^FE^) exhibited ROC AUC values exceeding 0.9 (p<0.001) in distinguishing active LN from inactive SLE, followed closely by calpastatin^FE^ and TFPI^FE^. Four of the FE ratios exhibited perfect sensitivity (calpastatin^FE^, PRDX6^FE^, PF4^FE^ and properdin^FE^), while ALCAM^FE^, PF4^FE^ and properdin^FE^ exhibited the highest specificity values for active LN. Overall, the three biomarkers exhibiting the highest ROC AUC statistic, sensitivity, and specificity values were ALCAM^FE^, PF4^FE^ and properdin^FE^, with the latter exhibiting perfect discrimination between active LN and inactive SLE, with 100% accuracy. As shown in **Table 3**, all biomarker FE ratios performed better than the conventional disease activity markers, anti-dsDNA, C3 and C4 (ROC AUC values: 0.63, 0.67 and 0.65, respectively) in detecting LN disease activity. At the other end of the spectrum were the serum levels of the proteins, which were generally poor in distinguishing active LN from inactive SLE.

**Table 3:** Diagnostic performance of serum, urine, and fractional excretion of biomarkers in active LN vs. inactive SLE.

Protein	AUC	95% confidence interval	Sens. (%)	Spec. (%)	AUC	95% confidence interval	Sens. (%)	Spec. (%)	AUC	95% confidence interval	Sens. (%)	Spec. (%)
	Active LN vs. inactive SLE (Urine)				Active LN vs. inactive SLE (FE)				Active LN vs. inactive SLE (Serum)			
ALCAM	0.84**	0.64 to 1.04	91.7	100	0.92***	0.79 to 1.06	91.7	91.7	0.65	0.42 to 0.87	50	83.3
Calpastatin	0.72	0.50 to 0.93	66.7	90	0.88*	0.69 to 1.06	100	75	0.57	0.28 to 0.86	44.4	85.7
Hemopexin	0.68	0.44 to 0.91	50	100	0.73	0.52 to 0.94	75	66.7	0.53	0.29 to 0.77	83.3	41.7
Peroxiredoxin-6	0.81**	0.63 to 1.00	75	100	0.88*	0.65 to 1.09	100	66.7	0.57	0.28 to 0.86	100	25
PF-4	0.94**	0.83 to 1.05	91.7	100	0.95***	0.85 to 1.05	100	91.7	0.68	0.46 to 0.90	75	66.7
Properdin	0.89**	0.77 to 1.03	91.7	100	1.0***	1.00 to 1.00	100	100	0.71	0.45 to 0.97	44.4	100
TFPI	0.83**	0.66 to 1.00	91.7	66.7	0.85**	0.68 to 1.01	91.7	75	0.80*	0.61 to 1.003	91.7	75
VCAM-1	0.90**	0.78 to 1.03	91.7	100	0.88**	0.73 to 1.01	83.3	83.3	0.83**	0.67 to 0.99	66.7	91.7
C3									0.67	0.43 to 0.89	91.7	50
C4									0.65	0.42 to 0.88	100	41.7
Anti-dsDNA									0.63	0.39 to 0.85	83.3	50
uPCR#	0.93***	0.82 to 1.01	91.7	100								

*p < 0.05; **p < 0.01; ***p < 0.001. Highlighted in red font are biomarkers where the FE metric outperformed the corresponding urine biomarker, in terms of test accuracy and statistical significance.

# It should be stressed that the new biomarkers should not be compared to uPCR for distinguishing active LN from other groups because uPCR is a major determinant of this clinical distinction, by definition.

For this analysis, the FE biomarker is regarded as having improved diagnostic performance if it exhibits increased ROC AUC accuracy value, with statistical significance of equal or higher value, compared to that of the urine biomarker. Indeed, the FE biomarker exhibited increased ROC AUC accuracy value, with statistical significance of equal or higher value, compared to that of the corresponding urine biomarker, in the case of ALCAM^FE^, PF4^FE^ and properdin^FE^, calpastatin^FE^ and TFPI^FE^, as indicated in red font in **Table 3**. Interestingly, use of the FE marker instead of the urine protein increased the sensitivity of detection of active LN in three cases (calpastatin^FE^, PF4^FE^ and properdin^FE^). The most dramatic improvement was seen with properdin, where the accuracy value increased from 0.89 (P<0.01) to 1.00 (P<0.001) when the FE ratio was used instead of the urine protein (**Table 3**).

Comparing the discriminatory potential of the urine:serum FE ratios against that of just the Cr-normalized urine and serum biomarkers in distinguishing active LN versus active non-renal lupus, as illustrated in **Supplementary Table 1**, properdin^FE^ exhibited the best performance (AUC 0.92, P<0.01), with excellent specificity (100%) and sensitivity (87.5%). Similar improvements in diagnostic accuracy were also noted when FE ratios were used to discriminate active LN from all other SLE patients (**Figure 5**). Again, properdin^FE^ showed superior performance (AUC 0.96, P<0.001), followed by ALCAM^FE^, calpastatin^FE^, PRX6^FE^, VCAM-1^FE^, and hemopexin^FE^.

### Validating the utility of FE ratios in identifying active LN in an independent validation cohort

In an independent validation cohort comprised of 25 active LN and 25 inactive SLE patients, we examined the top 3 biomarkers from the initial study (ALCAM, PF4, and properdin). As illustrated in **Table 4**, the AUCs of ALCAM^FE^, PF4^FE^ and properdin^FE^ surpassed corresponding urine and serum biomarker levels in predicting LN activity, validating our earlier findings. In this replication cohort, ALCAM^FE^ exhibited 95% accuracy in distinguishing active LN from inactive SLE, validating earlier findings and highlighting this as a novel biomarker for distinguishing active LN.

**Table 4:** Validation of FE ratios in an extended cohort of 50 LN patients.

Protein	AUC	95% confidence interval	AUC	95% confidence interval	AUC	95% confidence interval
	Urine Biomarker Protein Active LN vs. inactive SLE		Fractional Excretion Ratio Active LN vs. inactive SLE		Serum Biomarker Protein Active LN vs. inactive SLE	
**ALCAM**	0.92	0.83 - 1.01	0.95	0.90 - 1.00	0.55	0.38 - 0.71
**PF4**	0.74	0.60 - 0.88	0.75	0.62 - 0.89	0.66	0.50 - 0.81
**Properdin**	0.78	0.65 - 0.92	0.79	0.65 - 0.92	0.66	0.50 - 0.82

## Discussion

Fractional excretion is the fraction of a substance filtered by the kidney that is excreted in the urine. To provide an accurate picture of renal handling of a filtered molecule, the urine and plasma concentrations must be compared (16). In clinical practice, FE of sodium is commonly used as part of the evaluation of acute kidney failure. However, the efficacy of the test is best when used in conjunction with other clinical data. A few reports have also examined the utility of FE in chronic kidney disease (CKD). FE of sclerostin, MCP-1, and a couple of other proteins have been reported to be useful in assessing renal injury or monitoring chronic kidney disease (9, 17). Likewise, Deekajorndech (18) reported that FE of magnesium (Mg) was associated with the magnitude of tubulointerstitial fibrosis, whereas normal FE values were associated with intact tubulointerstitial structure, beating creatinine clearance. FE is influenced by at least two major determinants, including the renal tubular cells’ reabsorption capacity and the relative distribution of the molecule in question in relation to the tubular cell (18, 19).

This cross-sectional study represents the first analysis of FE ratios of urine biomarker proteins in SLE, using a cohort of 36 patients from whom both serum and urine samples were available. The FE ratios of all 8 proteins interrogated outperformed conventional disease activity markers such as anti-dsDNA, C3 and C4 in identifying renal disease activity. Renal SLEDAI (and PGA) scores correlated strongly with FE ratios of the assayed biomarkers, showing strong significant correlation with properdin^FE^, followed by ALCAM^FE^, calpastatin^FE^ and PRX6^FE^. Properdin^FE^, PF4^FE^, ALCAM^FE^, calpastatin^FE^, and TFPI^FE^ ratios exhibited the highest accuracy in distinguishing active LN from inactive SLE. Four of the FE ratios exhibited perfect sensitivity (calpastatin^FE^, PRDX6^FE^, PF4^FE^ and properdin^FE^), while ALCAM^FE^, PF4^FE^ and properdin^FE^ exhibited the highest specificity values for active LN. Overall, the three FE biomarkers exhibiting the highest ROC AUC c statistic, sensitivity, and specificity values for distinguishing active LN were ALCAM^FE^, PF4^FE^ and properdin^FE^.

The goal of this exploratory study was to determine if the FE biomarkers can outperform the corresponding urinary biomarker proteins. In this respect, it is noteworthy that the FE ratios exhibited increased ROC AUC accuracy value, with statistical significance of equal or higher value, compared to that of the corresponding urine biomarker, in the case of 5 of the eight markers interrogated, namely ALCAM^FE^, PF4^FE^, properdin^FE^, calpastatin^FE^ and TFPI^FE^. Two additional biomarkers, Hemopexin^FE^ and peroxiredoxin-6^FE^ also exhibited increases in ROC AUC values compared to the corresponding urine proteins, in discriminating active LN, though these were not associated with increased statistical significance. The observation that the accuracy values associated with seven of the eight markers tested increased when FE ratios were used call for further investigation of FE ratios as potential markers of active renal involvement in SLE. The exception to this rule was VCAM-1, whose levels in serum alone was discriminatory; indeed sVCAM1 was the strongest serum biomarker of active LN, alluding to the potential systemic origin and role of this adhesion molecule in disease.

In the present study, ALCAM^FE^ exhibited consistently high (>90%) accuracy, sensitivity, and specificity values in distinguishing active LN from active non-renal, inactive SLE and healthy controls, outperforming uALCAM. Of significance, in the validation cohort, ALCAM^FE^ exhibited 95% accuracy in distinguishing active LN from inactive SLE, validating earlier findings and highlighting this as a novel biomarker for distinguishing active LN. ALCAM^FE^ also revealed strong positive correlation with renal-SLEDAI and good correlation with SLEDAI, anti-dsDNA and PGA, as well as an association with higher renal pathology AI. This finding is in line with recent studies (8, 20–22) where urinary ALCAM was increased in active LN patients especially those with proliferative LN. ALCAM (CD166) is a glycoprotein and adhesion molecule of the immunoglobulin superfamily that is involved in T-cell co-stimulation, cell adhesion, angiogenesis, monocyte transmigration, and leukocyte intravasation into tissues (20, 23–26). Not surprisingly, this protein predicts renal function deterioration on long-term follow-up, and also exhibits a direct pathogenic role in LN, based on recent preclinical studies (20, 21).

PF4^FE^ exhibited outstanding accuracy (0.95), sensitivity (100%), and good specificity (>90%) in distinguishing active LN from other SLE, outperforming uPF4 as well as conventional disease activity markers such as anti-dsDNA, C3 and C4. PF4^FE^ also showed significant positive correlations with all SLE activity measures, and significant negative correlations with complement C3 and C4, and association with higher renal pathology AI. These findings are consistent with earlier reports reporting uPF4 as a promising biomarker for distinguishing active LN patients and for predicting biopsy activity changes in LN (3, 8, 27). PF4 functions in integrin-dependent mechanism controlling angiogenesis, promotes the expression of pro-fibrotic cytokines such as IL-4 and IL-13, enhances the proliferation of regulatory T cells, and plays a role in multiple diseases including systemic sclerosis and antiphospholipid syndrome (28–34). Whether PF4 plays a direct pathogenic role in LN remains unknown.

Properdin^FE^ exhibited perfect diagnostic metrics (100% accuracy, specificity and sensitivity) in distinguishing active LN from other SLE, and from inactive SLE, outperforming uProperdin as well as conventional disease activity markers such as anti-dsDNA, C3 and C4. Importantly, it showed the strongest positive correlation with renal SLEDAI (Rho 0.79, P< 0.0001), as well as an association with higher renal pathology AI. Properdin, a ~50 kDa plasma glycoprotein, is the only established positive regulator of the alternative pathway (AP) of complement activation *via* stabilizing C3 and C5 convertases, thus prolonging their half-life (35–39). Not surprisingly, targeting properdin, either genetically or pharmacologically, has been shown to effective in countering a number of renal diseases where properdin is elevated, including hemolytic uremic syndrome, ANCA-associated vasculitis, C3 glomerulopathy, IgA nephropathy, renal transplantation and murine model of acute anti-glomerular basement membrane disease (36–45). Recently, it has been shown that properdin-deficient MRL/lpr mice have reduced LN, again underscoring the pathogenic relevance of this molecule in this disease (46). Taken together with its outstanding diagnostic metrics, properdin warrants further study as a disease biomarker and potential therapeutic target in LN.

In summary, given that the urine:serum fractional excretion ratios of most of the urine biomarkers interrogated in this exploratory study outperformed corresponding urine protein measurements in identifying active LN, FE ratios ought to be systemically evaluated for their diagnostic utility in LN assessment. Specifically, the superior diagnostic metrics associated with ALCAM, PF4 and properdin FE ratios in distinguishing clinically active LN and higher renal pathology AI, warrant further investigation of these 3 proteins as potential harbingers of renal injury in SLE, and predictors of short-term and long-term renal function in LN. These same proteins may also hold promise in monitoring treatment response following induction therapy in LN. On the flip-side, FE calculations necessitate more assays to be performed (i.e., serum biomarker assays and Cr assays) and the supporting logistics to carefully collect paired urine/serum samples from the same patients at the same time. Also, the overlap of the confidence intervals for the ROC AUC accuracy values of the FE marker for ALCAM, calpastatin, PF4, Properdin and TFPI and their corresponding urine protein biomarkers raises the need for further validation of these novel metrics in independent cohorts and larger sample sizes, to ascertain if the FE ratios continue to show improved potential to distinguish active LN from inactive disease, compared to the corresponding urine proteins.

The limitations of this study include the limited cohort size and its cross-sectional nature. Despite including only 12 subjects per group, this study already underscores the diagnostic potential of FE biomarkers in LN, as several of these outperform the corresponding urinary protein biomarkers and conventional clinical measures (including proteinuria) in identifying clinical or renal pathology activity in LN. Furthermore, replication studies in the validation cohort substantiated findings in the initial discovery cohort, with ALCAM^FE^ exhibiting the highest diagnostic accuracy. Thus, this study will help guide future validation studies in larger cohorts, including subjects of multiple ethnicities. Finally, longitudinal studies are warranted to assess if ALCAM^FE^, PF4^FE^, properdin^FE^ can be used to track renal disease serially in LN or to track changes in renal pathology activity.

## Data availability statement

The original contributions presented in the study are included in the article/**Supplementary Material**. Further inquiries can be directed to the corresponding author.

## Ethics statement

The studies involving human participants were reviewed and approved by institutional review boards (IRBs) at the Tuen Mun Hospital, Hong Kong SAR, China and the University of Houston. The patients/participants provided their written informed consent to participate in this study.

## Author contributions

SAS, SS, and KV performed the experiments. SAS, KV, and FI performed the data analyses. CCM provided patient samples. CCM and CM designed the studies and reviewed all data. SAS, CCM, and CM wrote the manuscript. All authors reviewed the manuscript and concurred with the findings.

## Funding

This work is supported by NIH R01 AR074096.

## Acknowledgments

We acknowledge the statistical feedback and guidance provided by Drs. Lee and Pedroza on this manuscript.

## Conflict of interest

The authors declare that the research was conducted in the absence of any commercial or financial relationships that could be construed as a potential conflict of interest.

## Publisher’s note

All claims expressed in this article are solely those of the authors and do not necessarily represent those of their affiliated organizations, or those of the publisher, the editors and the reviewers. Any product that may be evaluated in this article, or claim that may be made by its manufacturer, is not guaranteed or endorsed by the publisher.
