# Supplementary material for: Exploring urine:serum fractional excretion ratios as potential biomarkers for lupus nephritis
Source: Front Immunol. 2022 Aug 24;13:910993. doi: 10.3389/fimmu.2022.910993 (PMC9449537; doi:10.3389/fimmu.2022.910993)
Supplement: Supplementary file 1 [file DataSheet_1.docx]

**Supplementary Table 1: Diagnostic performance of serum, urine, and fractional excretion of biomarkers in active LN vs. active non-renal SLE:**

| Spec. (%) | Sens. (%) | 95% confidence interval | AUC | Spec. (%) | Sens. (%) | 95% confidence interval | AUC | Spec. (%) | Sens. (%) | 95% confidence interval | AUC | Protein |
| --- | --- | --- | --- | --- | --- | --- | --- | --- | --- | --- | --- | --- |
| Active LN vs. non-renal SLE (Serum) | | | | **Active LN vs. non-renal SLE (FE)** | | | | **Active LN vs. non-renal SLE (Urine)** | | | |  |
| 83.3 | 41.7 | 0.31 to 0.79 | 0.55 | 91.7 | 91.7 | 0.76 to 1.05 | 0.91*** | 91.7 | 91.7 | 0.78 to 1.06 | 0.92*** | **ALCAM** |
| 62.5 | 100 | 0.58 to 1.04 | 0.81* | 100 | 62.5 | 0.70 to 1.05 | 0.88* | 75 | 83.3 | 0.66 to 0.99 | 0.83** | **Calpastatin** |
| 41.7 | 75 | 0.29 to 0.77 | 0.53 | 75 | 66.7 | 0.54 to 0.95 | 0.74* | 83.3 | 58.3 | 0.46 to 0.91 | 0.69 | **Hemopexin** |
| 75 | 50 | 0.21 to 0.79 | 0.50 | 50 | 100 | 0.53 to 1.07 | 0.80 | 75 | 83.3 | 0.63 to 0.98 | 0.81* | **Peroxiredoxin-6** |
| 91.7 | 33.3 | 0.46 to 0.89 | 0.68 | 83.3 | 75 | 0.52 to 0.96 | 0.74* | 75 | 91.7 | 0.66 to 1.01 | 0.84** | **PF-4** |
| 62.5 | 62.5 | 0.23 to 0.83 | 0.53 | 100 | 87.5 | 0.77 to 1.07 | 0.92** | 91.7 | 75 | 0.67 to 1.02 | 0.84** | **Properdin** |
| 66.7 | 91.7 | 0.55 to 0.97 | 0.76* | 75 | 100 | 0.75 to 1.02 | 0.89** | 83.3 | 91.7 | 0.79 to 1.04 | 0.92*** | **TFPI** |
| 100 | 25 | 0.28 to 0.77 | 0.52 | 100 | 41.7 | 0.57 to 0.95 | 0.76* | 66.7 | 66.7 | 0.49 to 0.92 | 0.71 | **VCAM-1** |

*p < 0.05; **p < 0.01; ***p < 0.001. Highlighted in red font are biomarkers where the FE metric outperformed the corresponding urine biomarker, in terms of test accuracy and statistical significance.

| **Supplementary Table 2. ELISA assay ranges of tested biomarkers** | | | |
| --- | --- | --- | --- |
| **Biomarker** | **Assay Range** |  |  |
| ALCAM | 62.5-4,000 pg/mL |  |  |
| Calpastatin | 0.25-8 ng/mL |  |  |
| Hemopexin | 6.25-200 ng/mL |  |  |
| PRX6 | 1.56-100 ng/mL |  |  |
| PF-4 | 15.63-1000 ng/mL |  |  |
| Properdin | 15.63-1000 ng/mL |  |  |
| TFPI | 15.6-1000 pg/mL |  |  |
| VCAM-1 | 15.6-1000 pg/mL |  |  |
